# Walking the Food Security Tightrope—Exploring the Experiences of Low-to-Middle Income Melbourne Households

**DOI:** 10.3390/ijerph15102206

**Published:** 2018-10-10

**Authors:** Sue Kleve, Sue Booth, Zoe E. Davidson, Claire Palermo

**Affiliations:** 1 Department of Nutrition, Dietetics and Food, School of Clinical Sciences, Faculty of Medicine, Nursing and Health Sciences, Monash University, Level 1, 264 Ferntree Gully Road, Notting Hill 3168, Australia; zoe.davidson@monash.edu (Z.E.D.); claire.palermo@monash.edu (C.P.); 2 College of Medicine and Public Health, Flinders University, GPO Box 2100, Adelaide 5000, Australia; sue.booth@flinders.edu.au

**Keywords:** food insecurity, low-to-middle income, experience, mixed methodology research

## Abstract

There is limited evidence of how Australian low-to-middle income (AUD $40,000–$80,000) households maintain food security. Using a sequential explanatory mixed methods methodology, this study explored and compared the food security (FS) and insecurity (FIS) experiences of these households. An initial quantitative survey categorised participants according to food security status (the 18-item United States Department of Agriculture Household Food Security Survey Module) and income level to identify and purposefully select participants to qualitatively explore food insecurity and security experiences. Of the total number of survey participants (*n* = 134), 42 were categorised as low-to-middle income. Of these, a subset of 16 participants (8 FIS and 8 FS) was selected, and each participant completed an in-depth interview. The interviews explored precursors, strategies to prevent or address food insecurity, and the implications of the experience. Interview data were analysed using a thematic analysis approach. Five themes emerged from the analysis: (i) food decision experiences, (ii) assets, (iii) triggers, (iv) activation of assets, and (v) consequences and emotion related to walking the food security tightrope. The leverage points across all themes were more volatile for FIS participants. Low-to-middle income Australians are facing the challenges of trying to maintain or improve their food security status, with similarities to those described in lower income groups, and should be included in approaches to prevent or address food insecurity.

## 1. Introduction

Food insecurity—the limited or uncertain availability of individuals’ and households’ physical, social, and economic access to sufficient, safe, nutritious, and culturally relevant food—is a complex, persistent, and multidimensional phenomenon [1]. Irrespective of an abundance of food and relative wealth, the issue of food insecurity is one experienced amongst high income countries, including Australia. The 2011–2012 National Health Survey, using a single-item tool, indicated that 4% of Australians, or approximately one million, were living in a household that was food insecure [2]. Utilising different valid multi-item tools, the prevalence of food insecurity in other high income countries was found to be 15% in New Zealand [3], 12.3% in Canada [4], 8% in England, Wales, and Northern Ireland (U.K.) [5], and 14% in the United States (U.S.) [6].

Food insecurity has a temporal dimension, and households may transition between episodic or chronic experiences [7]. The core characteristics of food insecurity have been described at both an individual and household level to include anxiety, concern, compromise to the quantity and nutritional quality of food, and social isolation [8,9]. The food insecurity experience may vary in severity along a continuum [10]. At one end of the continuum are initial indicators, such as anxiety and concern about an adequate food budget or food supply, and, at the other extreme end, the more severe indicators, perturbations in diet quality and quantity of food intake and hunger, become apparent [7,8,10,11]. Numerous negative implications of food insecurity have been reported, including physical, social, and emotional health impacts across the lifespan [12,13,14,15,16] and developmental and educational impacts in children [17]. Food insecurity is a serious public health issue.

Regardless of households’ geographic location, food insecurity is influenced by the interactions of a range of factors as described by the four dimensions of food security—food availability, supply, utilisation, and stability [1]—and the socio-demographic characteristics of households [3,18,19,20,21,22]. The major predictor of food insecurity is a low income or limited available economic resources for purchasing food or general resources in a household [18,19,23,24,25,26,27]. Although an inverse relationship between income and food insecurity exists [19,24,28], not all very-low-income households are food insecure, nor are households progressing up the income gradient food secure [28,29,30]. While the prevalence of food insecurity is greater in very-low-income groups, evidence from high-income countries indicates that households beyond this income group are experiencing food insecurity [19,26,31,32,33,34,35,36]. Categorisation of food insecurity based on the static measure of annual income may be problematic as this measure is insensitive to sudden economic changes within a household [28].

Whilst the existence of food insecurity in higher-income groups has been reported, there has been limited research examining the factors that contribute to food insecurity in these groups. Additional factors for Canadian and U.S. higher-income households include a fluctuating income, a sudden change in employment, a change in household composition, illness, disability, increased housing costs, and housing tenure [34,36,37]. Further significant predictors reported from Victoria, Australia in low-to-middle income households include an inability to raise money in an emergency, housing tenure, support from friends, and the cost of food [32].

There is a limited understanding of the nature of the experience of food insecurity in low-to-middle income Australian households. This may hinder the development of approaches to address the determinants of food insecurity more broadly across income groups. Furthermore, the factors that protect people from food insecurity and the coping strategies of households need to be explored. Approaches to address food insecurity need to consider the complex range of determinants that trigger food insecurity in households; and, consequently, a measurement of food insecurity must capture these determinants.

This study had three aims. The first was to identify low-to-middle income Melbourne participants who are food secure and food insecure. The second was to explore and compare food security and insecurity experiences; specifically, the precursors to, and strategies for preventing or addressing, food insecurity. The third was to examine the implications of the experience of food insecurity for those experiencing it to inform policy and practice.

## 2. Materials and Methods

### 2.1. Study Design

The study employed a pragmatic approach and positioning. The researchers were interested in understanding the experience of food insecurity from the perspective of participants from low-to-middle income households and the implications of this on their lives for policy and practice. An explanatory sequential mixed methods research design approach of collecting, analysing, and integrating both quantitative and qualitative data in the research process was employed [38,39,40]. Typically, the emphasis in this design is on the quantitative phase; however, in this study, the research emphasis was on the qualitative phase to explore the experience of food security and food insecurity within low-to-middle income households. The initial quantitative results were used to identify and purposefully select participants to qualitatively examine the food insecurity phenomenon [38,41].

The study was conducted according to guidelines in the Declaration of Helsinki, and all procedures were approved by the Monash University Human Research Ethics Committee (CF14/1382-201400647). Informed consent was implied for the quantitative phase, and written informed consent was obtained for the qualitative phase.

### 2.2. Participants

A cross-sectional convenience sample was recruited from metropolitan Melbourne, Victoria. Suburbs were selected according to the ‘Vulnerability Assessment for Mortgage, Petrol, and Inflation Risks and Expenditure’ (VAMPIRE) 2008 Index [42]. The VAMPIRE index is based on Census data and calculates suburb vulnerability based on three socio economic stressors: mortgage, car, and income, providing a ranking from minimal to very high vulnerability. Those with high levels of car ownership, who journey to work by car, who have mortgage tenure, and/or who have low incomes are considered ‘more vulnerable’. A higher vulnerability VAMPIRE rating is likely to impact on finances available for food [43]; thus, all Melbourne suburbs with medium to very high ratings were selected for inclusion. These suburbs provided a varied sample in which food insecurity is likely to occur in some households due to characteristic stressors [44].

**Figure 1:**
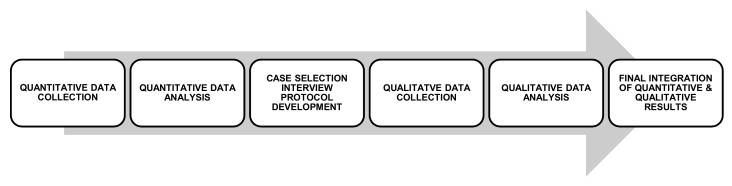
Summary of Sequential Explanatory Mixed Methods research design.

The convenience sample aimed to identify information-rich participants to interview as part of the qualitative phase, rather than be representative of the population. Eligibility for study inclusion was conducted in two stages. In the quantitative phase, participants were over 18 years of age and residing in metropolitan Melbourne, living in or adjacent to VAMPIRE suburbs. In the qualitative phase, participants from the quantitative phase were included as low-to-middle income if they had a gross household income of AUD $40,000–$80,000 per annum before tax. This income categorisation was based on Australian Bureau of Statistics quintiles of gross Victorian household income [45]. Respondent anonymity was preserved by a unique code that was assigned for survey responses, and all interview participants were provided with a pseudonym. Figure 1 summarises the study design procedures.

### 2.3. Quantitative Phase: Data Collection and Analysis

The quantitative survey, ‘Food Security in Melbourne Households Survey’ (FSiMH survey), was designed by the researchers using a mix of validated questions and instruments. Demographic questions were developed to gather information on factors that are associated with food insecurity in the literature and support categorisation based on income [19,32,46]. Food security status was determined using the validated 18-item United States Department of Agriculture Household Food Security Survey Module (USDA-HFSSM) [7]. The survey was promoted across a diverse range of community organisations and websites located in, or in close proximity to, the VAMPIRE suburbs. The main household shopper or food preparer was asked to complete the survey. The FSiMH survey was administered in both an electronic (Qualtrics, Provo UT, US platform) and paper format between September 2014 and February 2015.

The USDA-HFSSM was selected for determination of food security status because of its reliability across populations and population subgroups and its ability to capture the severity level and continuum of experience of food insecurity [7,47,48,49]. The USDA-HFSSM categorises households as food secure or food insecure with varying severity levels of experience. Households with affirmative scores of 0–2 are classified as food secure; those with an affirmative score of 0 are classified as food secure at the high food security (no reported indications of food-access limitations) severity level, whereas those with affirmative scores of 1 or 2 are classified as food secure at the marginal food security (anxiety over food sufficiency or a shortage of food in the house) severity level. Scores of 3 or greater are classified as food insecure at the low food security (reduced quality and variety of food with little or no indication of reduced intake) and very low food security (multiple indications of a disrupted eating pattern and reduced food intake) severity levels [7]. Studies from the United States and Canada report an increase in marginally food secure households that display greater health outcomes and similar characteristics to food insecure households [4,10,24,50]. Those who are marginally food secure may also be at greater risk of progressing to more severe forms of food insecurity. Consequently, using the philosophical pragmatic approach that guides this research, the modified Canadian food security categorisation was applied [4]. Respondents that were classified as experiencing marginal food security with a score of 1 or 2 were included in the food insecure category. The severity categorisations and scores are consistent with the USDA-HFSSM classifications [7,51].

Data were analysed using the statistical software package IBM SPSS Statistics for Windows, Version 22.0 (SPSS INC., Chicago, IL, USA). For the purpose of this analysis, respondents were dichotomised as food secure or food insecure, and demographic characteristics were explored descriptively and reported as counts and percentages.

### 2.4. Qualitative Phase: Data Collection and Analysis

The results from the quantitative phase supported the case selection and the interview protocol’s development. The logic underpinning the interview protocol and questions was informed by both the existing literature [9,25,52,53,54] and the quantitative analysis, in particular the responses to the USDA-HFSSM items that described the experiences and consequences of food insecurity. The USDA-HFSSM assesses food security status based on an inability to access food due to a lack of financial resources; however, additional factors beyond this may impact upon food security status [47,55]. Consequently, the interviews allowed for elaboration and exploration beyond these economic factors and a deeper understanding and extension of the experiences of food insecurity that are measured by the USDA-HFSSM questions. The interviews explored low-to-middle income participants’ experiences of accessing food (physical and economic), factors that influenced and impacted this, and the consequences of these factors. Four key areas were explored in the interviews: (i) accessing food and food choices for the household, (ii) factors impacting on food for the household, (iii) consequences when sufficient food quantity and preferred foods cannot be accessed, and (iv) coping and protective strategies: asset exploration (Supplementary Table S1). The researcher used a semi-structured interview format whereby the key areas were used to construct the main questions that were asked of participants and a series of prompting questions that were subsequently asked based on participants’ initial response. The interviewer continued probing the participants until they were satisfied that responses of an adequate breadth and depth in each of the four interview areas were obtained.

All interviews were individually undertaken between June 2015 and September 2015 by the first author with each participant at a mutually suitable time in interview rooms at local community centres. The interviews were digitally recorded and transcribed, and field notes were kept after each interview. The interview duration ranged from 45 to 90 min. The NVivo qualitative software (QSR International, Version 10.3, Melbourne, Australia) was used to manage, store, and support the data analysis. A thematic data analysis was chosen, as the researchers acknowledged the complexities of food security and the need for more than one theoretical framework to explain the data and the emergence of new concepts. Braun and Clarke (2006) describe the benefits of a thematic analysis as ‘providing a flexible and useful research tool, which can potentially provide a rich and detailed, yet complex account of data’ [56]. The qualitative analysis approach included familiarisation with a transcript’s content, open content coding with coding nodes, and inter-coder agreement. The codes were grouped into themes and subthemes in light of the research questions with the verification of themes amongst the researchers. A constant comparison approach to analysis was performed to describe patterns in the data to inform the initial formation of categories, where a content comparison within each category enabled the description of categories to evolve [57,58]. The constant comparison approach was implemented at three levels: for individual participants regardless of food security status; within food secure and food insecure groups; and between food secure and food insecure groups [57]. This analysis approach allowed for exploration of similarities and differences across and between groups.

## 3. Results

### 3.1. Quantitative Phase: Demographic Characteristics and Food Security Status

One hundred and thirty-four participants completed the FSiMH survey. Thirteen participants declined to indicate their income level, reducing the participant income data to *n* = 121. Forty-two participants were classified as low-to-middle income (food secure (FS), *n* = 26 and food insecure (FIS), *n* = 16), including 12 households with children.

The majority of participants were female and Australian-born. FIS participants (*n* = 16) included participants that were homeowners with a mortgage (*n* = 8), participants living with their spouse/partner and children (*n* = 11), and participants that had some form of paid employment (*n* = 11) (Table 1). In comparison, FS participants (*n* = 26) included participants that were homeowners (*n* = 19), of which nine were mortgage free, participants living with their spouse/partner and children (*n* = 10), and participants that had some form of paid employment (*n* = 10).

Twenty-four low-to-middle-income participants, FS (*n* = 12) and FIS (*n* = 12), consented in the FSiMH survey to be contacted to participate in the qualitative phase. Eight participants declined due to an illness, a work commitment, or no longer being interested in further participation. Sixteen in-depth interviews, FS (*n* = 8) and FIS (*n* = 8), were completed, 13 face-to-face and 3 by telephone. A key emphasis of qualitative research is the focus on the quality and not the quantity of interviews; so, sampling for the qualitative interviews in this study continued until theoretical data saturation was achieved. Theoretical data saturation in this study meant that the researcher was satisfied with the quality of the information that was obtained to be able to answer the research questions [54]. The majority of interview participants were female (*n* = 15), and nine were living in households with children. The most common housing tenure included mortgage holders (*n* = 11), and four participants were privately renting.

The severity of food insecurity experienced by the qualitative interview participants (*n* = 16) varied: marginal food security (*n* = 4, two with children), low food security (*n* = 2, one with children), and very low food security (*n* = 2, both with children).

### 3.2. Qualitative Results

The qualitative interview data analysis yielded 5 interacting themes and 10 subthemes. Table 2 summarises the key similarities and differences between and across the food-secure and food-insecure participants. The five main themes are presented below.

#### 3.2.1. Theme 1: Food Decisions are Complex, Dynamic, and Multi-Factorial

Irrespective of food security status, food decisions were complex, often interconnected, dynamic, and multifactorial in nature, with an array of influencing factors. The role and values that were associated with food, and internal factors, such as food budgets, were impacting factors. External factors, such as cost of food and food availability, also contributed to the dynamic nature of food decisions.

Food was a priority, as described by a FS participant, Amelia, who had experienced food insecurity growing up and stated that she ‘*would go without anything to make sure that food was on the table’* for her children. For those experiencing, or were at risk of, food insecurity, there were additional, often constant, pressures on food decisions for the household, where the complexity and interaction of deciding factors were magnified.

Both participant groups identified food as a social conduit that provided a connection to a community. However, this was described with some preoccupation by FIS participants, who detailed a more stressed approach to eating out or entertaining that impacted on their enjoyment of the social interaction when compared to FS participants:

*‘I try to avoid it. Most I’ll have is a coffee from uni …if they (Uni friends) buy lunch …. you miss out, but—there are times when I was really hungry and I didn’t have my lunch, so I had to buy it. That would mean…, ‘what am I going to do about that money when I shop on the weekend?’* Ann (FIS)

In contrast, FS participants described food as a medium to socialise over, with a greater sense of ‘freedom’ that enables social situations. This in part was reported to be influenced by a greater available budget that provided flexibility, the participant’s life stage, and the presence of children in the household.

Importance was placed on the quality and variety of nutritious foods. This value was often challenged for FIS participants, especially when the budget was tight, creating competing demands for the food dollar. Both groups of participants described a hierarchy of food decision drivers in which household bills were prioritised, impacting on the available food budget:

*‘meet my expenses first, and then what money I have left over is what I would do the shopping with. I think I’ve just stayed that way.’* Maureen (FIS)

Time was an important resource in food decisions for all participants, particularly when the main food gatekeeper worked, studied, and/or cared for children. Shopping and food preparation tasks were often time-consuming and labour-intensive. These tasks required high levels of organisation, and often impacted on decisions that were associated with foods that were purchased for convenience (for example, the use of pre-prepared vegetables) and the question of where to shop (for example, a supermarket versus a mix of shops). FIS participants reported investing a large amount of time and energy in shopping routines. A trade-off and compromise was described:

*’one of the biggest things that I think a lot of people have trouble with; is time … So it might be saving a little bit of money, but then it’s costing time, and time is probably more expensive now than that’* Ava (FS) and *‘it’s not easy to be able to spend money on whatever you want kind of thing, so I had to invest time to look around and shop around.’* Ann (FIS).

This highlights the difference in how each of the two participant types perceived time as a resource.

#### 3.2.2. Theme 2: Multiple Protective Assets

The participants described an array of skills and strategies that were used to both protect and support food security. Food literacy and social connections were assets that could be enacted, especially in times of greater need. For FIS participants, assets (financial, human, social, physical, and natural) were of greater intensity, well-developed, and varied. All participants described these food literacy ‘life skills’ as invaluable, with their development varying over each participant’s lifespan.

All participants described an array of financial management assets that were employed to manage food. The intensity of these skills was greatest for FIS participants. Some FS participants recalled life stages when fiscal resources were constrained. The management strategies that they used closely mirrored those that were used by FIS participants. FS participants described the importance of an overall budget to their household. However, how it was used varied significantly in FIS participants, where the budget was closely scrutinized, as Clara explains:

*‘depends on robbing Peter to pay Paul with the food budget… it goes down to the last $10 by the end of the week... what level of food we get for the week’* Clara (FIS)

Both FS and FIS participants described a range of practical strategies; for example, planning for and organisation of food to support money saving and to have pantry staples. Aspects of Theme 1 overlay this range of practical strategies.

Broader connections to community were evident across both FIS and FS participants, and were reported to be protective against food insecurity. An example is neighbours looking out for each other and sharing home grown produce. Social support that was provided by family and friends was evident. This was often in the form of general groceries and food, including meals.

#### 3.2.3. Theme 3: Food Insecurity Triggers Act Alone or Are Cumulative and May Be beyond Household Control

The food insecurity ‘triggers’ were often unforeseen events or experiences that impacted on food security status and were either internal or external to the participant’s household. Internal triggers included income changes, expected and unexpected expenses, and household composition changes. External triggers often reflected the broader system, economic situation, and food supply. All participants reported that these triggers acted alone or in unison, magnifying their effect on each other. Triggers, real or potential, were perceived to hover in the background of day-to-day life for FIS participants and were commonly reported. Triggers impacting on the household budget and/or total finances were points of stress and heightened the risk of food insecurity. However, participants from FS households, especially those with children, said that they were often still ‘*walking a budget tightrope’* Ava (FS). Those classified as food secure reported previous episodes where they had difficulties accessing food as a result of the reported food insecurity triggers. These experiences were detailed with evidence of anxiety and ‘*not wanting to go back there (being food insecure)*’Amelia (FS).

The financial triggers described by both FS and FIS participants were reported to manifest in a number of forms, from a sudden and unexpected reduction in household income or a change in household composition (birth of a child) to unexpected household expenses, including an increase living and medical expenses. These impacted on the financial stability and well-being of households, and influenced decisions on the question of whether the main caregiver should return to employment to relieve the financial load:

*‘No longer did we have additional income, bills kept coming plus the mortgage things were very tight.’* Ann (FIS) and *‘When my wife stopped working, we nearly went broke. We were down to our last dollar.’* Eric (FS)

Two FS participants without a car identified difficulties in easily accessing food due to limited public transport infrastructure in their area despite adequate financial resources.

#### 3.2.4. Theme 4: Assets Amplified: Juggling and Applying Management Strategies as Required

Whilst the assets described in Theme 2 were ever-present for all participants, it was not until one or more of the triggers (Theme 3) occurred that the assets were transformed and amplified into coping strategies. For FIS participants, there was a distinct difference in the rate and urgency of transformation of these assets. Often, these management strategies did not occur in isolation but in unison or in a staged format. This process of putting these assets into action could occur with or without support from the participant’s immediate household.

Saving money was recognised as an important strategy for all participants. For FIS participants, this was invariably difficult; it meant that there was never a reserve or buffer to draw upon. In contrast, most FS participants had at least one option as a backup plan if finances were limited, including savings, credit cards, and loan redraws. This was a key point of difference when compared to FIS participants who did not have these options:

*‘There are times when we have had to redraw on our home loan to have more money to live off... to buy food but sometimes the usual savings account may be down so we use Visa—that’s how we manage our money—then pay the card off at the end of the month so we never have to pay interest.’* Rowena (FS)

When finances were limited, alternative funding for shopping was enacted, including supermarket reward and loyalty schemes that allow cash/credit for shopping, by both FS and FIS participants:

*‘We have [Loyalty scheme name], quite often, it will be, ‘Do I need to convert my [Loyalty] points to [Loyalty] dollars, and can we go to [named Supermarket] and spend $10 getting what we need?’ I always leave that as my backup of the backup plan.’* Clara (FIS)

Both participant types discussed how such strategies often meant spending more on food or other household items that impact on food budgets in the short term. However, the long-term benefit of credit towards future shopping outweighed this short-term risk.

#### 3.2.5. Theme 5: The Consequences and Emotional Rollercoaster of Food Access and Provision

The consequences and emotions that were associated with food access and provision varied considerably. For FIS participants, the experience was often fraught with relentless emotional lows. The reported consequences of not being able to access food ranged from worry to compromises on food choices and amounts. Food-secure participants reflected on a significant past experience that was related to financial difficulties that impacted on food access and provision and instigated a range of emotions. Whilst stress and anxiety were evident for some FS participants, it was not to the extent described by FIS participants. However, the impact of these past experiences was significant enough for FS participants to reflect and articulate why they wanted things to be different:

*‘The juggle and stress to make ends meet was too much I deferred for a year, worked fulltime, earnt money, then went back the following year and completed my degree. I don’t want to go back to that stress.’* Lucy (FS)

Whilst the stress of food provision often dominated participants’ stories, there were also elements of triumph that were centred on respect, resilience, responsibility, and resourcefulness.

For both FIS and FS participants, respect, resilience, and resourcefulness grew from difficult experiences during childhood and adolescence:

*‘I’m a… stronger person because of my childhood: a person with a different upbringing may look at things differently.’* Clara (FIS) and

*‘It was really hard growing up and moving around all the time. Family is everything to me; it means stability, and I’m the rock for the family now… having them over for a meal helps this…’* Amelia (FS)

These experiences often shaped their current food access and provision life skills.

## 4. Discussion

The purpose of this study was to identify low-to-middle income food secure and food insecure households from Melbourne and explore and compare food security and insecurity experiences and implications. The results highlight the precarious nature of achieving food security in lower-income groups and the resourcefulness, resilience, and array of assets or strengths that participants use when facing triggers that threaten their food security. Furthermore, they indicate that those who were categorised as food secure using the USDA-HFSSM may be at risk due to the existence of additional factors beyond those of a financial origin, such as a lack of physical access to, or a limited supply of, culturally appropriate foods. To our knowledge, this is the first study to explore the experience of food insecurity of low-to-middle income households in Australia.

### 4.1. Low-to-Middle Income Households’ Experiences: Assets, Resourcefulness, Resilience, and Emotions

The food insecurity triggers that were described by both groups of participants, such as a change in income, increased cost of living expenses, and changes in household composition, are consistent with those reported for low-to-middle income households in Canada and the U.S. [34,36] and lower-income U.S. and Australian households [25,59,60]. The key differences between food-secure and food-insecure participants were the number and complexity of factors and the cumulative and relentless nature of the triggers.

The interviews allowed for the exploration of the range of assets possessed by both FS and FIS low-to middle income participants. At the core of these assets was food literacy and social connection, which supported both the capabilities and resources of the household. The existence of assets and skills inclusive of, but not limited to, budgeting and planning for food, and purchasing and preparing food, have been reported in food-insecure, lower-income households [52,61,62,63]. A key difference between FS and FIS participants in this study was the amplification of these assets and their ability to provide a crucial buffer to the food insecurity experience, but only up to a certain point. This is consistent with the limited capacity of food literacy skills to ameliorate the food insecurity experience because of the complex range of food insecurity determinants [64,65]. The range of assets was found to support the high degree of resourcefulness with food acquisition and (food and financial) management that was demonstrated by FS and FIS participants. The resourcefulness of individuals facing food insecurity has been reported previously, and should be considered in approaches to prevent or address food insecurity [63,64].

The asset of support was important to both FS and FIS participants. Social support in the food security literature has been described in the contexts of emotional, instrumental (child care, food, or material items), and informational support (advice and factual information) [66]. Consistent with this literature, the social support that is reported in this study was described as arising from two sources: (1) networks of family and friends, and (2) networks in the broader environment, such as community agencies and government benefits systems. Both FS and FIS participants described sourcing support predominately from friends and family and limited interaction with community welfare. This was driven by the potential shame and stigma, and confirms that reported in some low-income groups [63].

The associated emotions and experiences of trying to achieve or maintain food security were evident in both participant groups. Despite previous and current food insecurity experiences, its impacts were felt both psychologically and physically. Participants detailed the stress, shame, embarrassment, and concern due to the stigma of not being able to pay for food and/or feed children. The emotional experiences of these low-to-middle income participants are consistent with those reported principally by women in Australian and Canadian low-income, food-insecure households [9,25,63]. Often, counteracting these emotions was the high degree of resilience present in many participants. Resilience is a dynamic concept influenced by life-course events, and has been believed to contain two key elements: adversity and positive adaptation [67,68]. The level of resilience evident in both FS and FIS participants was shaped through life experiences that were often adverse in nature [69].

### 4.2. Categorisation of Food Security and Examining Etiology

The USDA HFSSM classification of food insecurity is based on a lack of money available to purchase food, and the interviews confirmed that financial factors/stressors were the main food insecurity trigger in the participant groups. While this finding supports the association with financial factors that has been described in the literature, it is important to reflect upon this trigger more broadly in the context of both financial constraints and assets [52,70]. The finding provides a rationale for examining the financial causes of food insecurity beyond household annual income, which is a static, insensitive measure and may not reflect sudden household economic changes that can temporarily lead to bouts of food insecurity [28,71]. Of note is that all low-to-middle income participants’ main income sources were from salaries alone, in some cases supplemented with Government assistance payments, such as the Family Tax Benefit. This is supported by previous studies that found that those who are employed also experience food insecurity [24,50,72,73]. Employment status, in particular having multiple part-time jobs rather than full-time work, has been associated with an increased risk of food insecurity [73]. Additionally, having more than one income earner in a household has been shown to reduce the odds of experiencing food insecurity [72]. In this study, 12 of the 16 interviewed participants indicated that the primary income earner in the household was employed at a full-time or near full-time level. Furthermore, in seven of these households, another member was employed full-time, part-time, or casually.

The participants discussed the need for sufficient income or financial resources to meet the rising cost of living expenses. The capacity to have savings available when needed was described by both FS and FIS participants as a crucial strategy to buffer against the impact of unexpected expenses, but one that some FIS participants described as being difficult to implement. The evidence for savings as a protective factor against food insecurity is recognised both internationally [74] and nationally [22,75]. Australian evidence on the association between the capacity to save and food insecurity is limited. Foley (2010) reported that those Australians who were unable to save were 6.5 times more likely to have experienced food insecurity in the last 12 months [75].

This research highlights two points related to food security status classification that warrant further consideration.

#### 4.2.1. Marginal Food Security Severity Categorisation

This study modified the food security classification from that of the original USDA-HFSSM protocol, where one or two affirmative responses were classified as food insecure at the severity level of marginally food secure, and allowed for exploration of their experience [10,14,24]. Understanding the marginally food secure experience has importance from epidemiological, public health, and public policy perspectives [14]. The decision to categorise those participants that were experiencing marginal food security as food insecure was supported by the findings, particularly by those stories that portrayed the experience of anxiety and stress regarding food provision. Despite two FIS participants being classified as marginally food insecure, their stories revealed a history of more severe forms of food insecurity over their lifetime and described rapid transitions between severity levels. As suggested by Loopstra (2013), those experiencing marginal food security may experience poorer health outcomes and increased forms of material hardship when compared to food-secure individuals [50].

#### 4.2.2. Classification of Food Security Status beyond Financial Resource Constraints

Whilst financial resource challenges may be the primary determinant of food security status, there may be circumstances where other determinants beyond this are challenged. The USDA-HFSSM is based on economic access to food; it does not take into consideration other reasons for the existence of food insecurity. A recent systematic literature review indicated that there is an absence of multi-item tools that can assess food security beyond the one dimension of financial access [47]. Both FS and FIS participants described additional experiences beyond those of financial resources that challenged their food security status and constituted limitations in their physical access to a food supply. For FS participants, this was despite having adequate financial resources. One FS participant described her recent move from interstate to an area that had poor public transport infrastructure, and, as she did not have a car, this resulted in a limited capacity to source culturally relevant foods. Despite being able to access some food in a small but more expensive food outlet, her food choices were compromised. A lack of access to a car has been associated with an increased difficulty of accessing food outlets [19,76]. This experience highlights the importance of all dimensions of food security, including an adequate supply, physical and economic access, and the resources to utilise food, to achieve and maintain food security [1], and supports the need for a food security measurement tool that is inclusive of these dimensions. Such a measurement tool, the Household Food and Nutrition Security Survey (HFNSS), which is based on the USDA-HFSSM, has been developed and undergone preliminary validation in Australia [77,78].

### 4.3. Strengths, Limitations, and Further Research

This study is the first Australian study to examine the existence and experience of FS and FIS in low-to-middle income Australian households. Additionally, the focus of the research in the qualitative phase provides an important contribution to the literature, particularly in Australia, as it provides the first exploration of the experience of food insecurity within this income group. The mixed-methods approach allowed for detailed exploration of the experiences of food insecurity and food security. The methodology supported the understanding of the construct and experience of food insecurity in this income group more than a quantitative or qualitative methodology alone. The constant comparison approach to the analysis supported the interpretation of the findings. An additional strength was the case selection method for the interviews, which supported the transferability of the qualitative findings. Selecting participants from those that had participated in the quantitative survey allowed for further interpretation of the findings when supported by the stories of participants.

A potential limitation is the gender-biased nature of the recruitment. This resulted from the main food provider completing the survey, which resulted in a higher number of women participants (88%). Fifteen women and one male were interviewed, which potentially may impact on the credibility and dependability of the interview data. The inclusion of only one male voice provided a narrow view of how men may perceive food insecurity. However, this response rate is reflective of gender food provision roles, where women predominantly have the responsibility of being the principal food provider [79], which may subsequently affect how they report these experiences. While a theoretical gender lens was not applied in this study, the findings on the physical, social, and emotional food insecurity experiences of women have been previously described in food-insecure households [80].

Further exploration of the experiences of, and the role of the extensive range of assets in, these low-to-middle income participants can better inform responses to food insecurity. In addition, more research is needed to explore the experience of food insecurity in different contexts, including: geographic locations of rural and metropolitan areas of Australia, sub-population groups, and both lower- and higher-income groups. This should include the exploration of determinants inclusive of a range of financial indicators, such as capacity to save, but also additional determinants of food insecurity. The use of mixed methods in future research efforts is crucial to provide a more detailed and rich understanding of the true and precarious nature of this phenomenon.

## 5. Conclusions

This study reveals novel and important findings on the existence of food insecurity amongst low-to-middle income Melbourne households, an income group that would not necessarily be considered food insecure within the context of a high-income country. Additionally, these findings support the precarious nature and balancing act of achieving food security for some low-to-middle income households. The experiences of those classified as marginally food secure confirm the need for further research within this severity-level group regardless of income.

While limited financial resources are a primary determinant of food security status, this research confirmed that there are multiple additional determinants that must be considered to maintain food security.. The results revealed the constant balancing act, especially of a range of financial, social, physical, and personal assets, that must be undertaken to prevent or alleviate the experiences of food insecurity. The findings of this work may be used to support policies and practices to prevent or alleviate food insecurity in low-to-middle income groups in urban Australia.

## Figures and Tables

**Table 1:** Characteristics of low-to-middle income survey respondents (*n* = 42) and in-depth interview participants (*n* = 16) according to food security status.

Demographic Characteristics	Quantitative Survey Respondents *n* = 42		Respondents Selected for Qualitative Interview *n* = 16	
	Food Insecure *n* = 16(%)	Food Secure *n* = 26(%)	Food Insecure *n* = 8(%)	Food Secure *n* = 8(%)
**Gender**				
Male	1(6.2)	4(15.4)	0	1(12.5)
Female	15(93.8)	21(80.8)	8(100.0)	7(87.5)
Prefer not to say	0	1(3.9)	-	-
**Age**				
18–25	2(12.5)	2(7.7)	1(12.5)	1(12.5)
26–35	6(37.5)	4(15.4)	2(25.0)	2(25.0)
36–45	5(31.3)	7(26.9)	3(37.5)	1(12.5)
46–55	1(6.2)	6(23.0)	0	3(37.5)
56–65	2(12.5)	3(11.5)	2(25.0)	0
Over 65	0	4(15.4)	0	1(12.5)
**Country of Birth**				
Australia	11(69.0)	16(61.5)	5(62.5)	4(50.0)
Other	5(31.0)	10(38.5)	3(37.5)	4(50.0)
**Housing Tenure**				
Homeowner, mortgage	8(50.0)	10(38.5)	4(50.0)	3(37.5)
Homeowner, no mortgage	0	9(34.6)	1(12.5)	3(37.5)
Renting, privately	8(50.0)	4(15.4)	3(37.5)	1(12.5)
Other	0	3(11.5)	0	1(12.5)
**Household Structure/Composition**				
Living alone	1(6.2)	1(3.9)	2(25.0)	0
With parents/family	0	3(11.5)	1(12.5)	1(12.5)
With spouse/partner	1(6.2)	11(42.3)	1(12.5)	3(37.5)
With spouse/partner and children <18 years	10(62.5)	10(38.5)	4(50)	3(37.5)
With spouse/partner and children >18 years	1(6.2)	0	0	1(12.5)
With my children <18 years	2(12.5)	1(3.9)	0	0
Living in a share house	1(6.2)	0	0	0
**Number of children in household**				
0	4(25.0)	14(53.9)	3(37.5)	4(50.0)
1	3(18.8)	3(11.5)	1(12.5)	1(12.5)
2	8(50)	4(15.4)	3(37.5)	3(37.5)
3	1(6.2)	5(19.2)	1(12.5)	0
**Education Level Attained**				
Completed some school	4(25.0)	7(26.9)	2(25.0)	2(25.0)
Completed school	1(6.2)	2(7.7)	2(25.0)	1(12.5)
TAFE ^1^, diploma, or trade	6(37.5)	5(19.2)	0	1(12.5)
Any completed tertiary study	5(31.3)	12(46.2)	4(50.0)	4(50.0)
**Employment**				
Full-time paid work	4(25.0)	3(11.5)	2(25.0)	2(25.0)
Part-time paid work	3(18.8)	4(15.4)	0	1(12.5)
Casual paid work	3(18.8)	2(7.7)	1(12.5)	0
Work without pay (family business)	1(6.2)	1(3.9)	1(12.5)	3(37.5)
Home duties	3(18.8)	7(26.9)	1(12.5)	0
Unemployed	0	2(7.7)	0	0
Studying	2(12.5)	1(3.9)	0	0
Studying + casual/part time work	*	*	3(37.5)	1(12.5)
Studying + house duties	*	*	1(12.5)	0
Carer	0	1(3.9)	0	0
Retired	0	5(19.2)	0	1(12.5)
**Income source**				
Salary	*	*	5(62.5)	4(50)
Salary and Government benefit	*	*	3(37.5)	2(25.0)
Savings and Superannuation	*	*	0	1(12.5)
Savings and Government benefit	*	*	0	1(12.5)
**Main Transport**				
Car/Motor Bike	14(87.5)	24(92.3)	6(75.0)	8(100.0)
Walking/Bike	2(12.5)	0	1(12.5)	0
Public Transport	0	2(7.7)	1(12.5)	0

* Not collected in the Food Security in Melbourne Households (FSiMH) survey. ^1^ TAFE, Technical and Further Education.

**Table 2:** Summary of theme and subtheme comparison between and across the food-secure and food-insecure participants.

Themes and Sub Themes	Both Food-Secure & Food-Insecure Participants	Food-Secure Participants	Food-Insecure Participants
**Theme 1: Food decisions are complex, dynamic, and multi-factorial**			
**Roles and values that shape food decisions**	Food provision is a priority especially if children are present but money available for food challenges this.	Greater freedom for social eating but less likely to eat out with children due to cost.	Food is the priority but this is a challenge when the budget is pressured
	Food provides a connection to a community.		Stress related to social eating: budget manipulation required. Dilemmas created and potential ramifications.
**Other forces that shape household food decisions**	Nutrition/health priority: Quality and variety	Cognisant of food ethics: supermarket duopoly. Some households’ greater financial capacity: able to respond	Budget tightrope: constant compromises to food choices
	Time available to cook and shop		
**Theme 2: Multiple protective assets: financial, social, physical, human, natural**			
**Strength in food literacy capabilities and resources**	Food literacy skills/resourcefulness	*	Amplification of resourcefulness and food literacy skills. Budget assets are highly refined, creative, time-consuming, and may be unique to the household but are in a constant state of play at greater intensity. Food cost literacy: developed capabilities to monitor food costs; with product knowledge
	Budgeting skills and strategies are defined but have a differing intensity level across all households		
	Highly refined planning, food preparation, shopping assets		
	Knowledge of food alternatives: supporting modifications to food for the household.		
	Resourcefulness present and developed based on life experiences.		
**Strength in social capital capabilities and resources**	Connection to community/agencies that is required to know what broader financial resources are possible.	*	Connections to the broader community and social support from family and friends; these relationship assets support other assets or may facilitate them to action. Greater sense of resilience drawn from within based on personal experiences and at times less reliance on social relationships
	Communities look out for each other		
	Relationships to support food literacy skills within and external to households: role models		
	Growing food facilitates relationships with neighbours/community		
**Theme 3: Food insecurity triggers act alone or are cumulative and may be beyond household control**			
**Internal triggers**	Time available to shop and cook can manifest in households in different ways	Episodic nature of triggers. Households may have experienced triggers in past life stages that increase the risk of food insecurity; these were recalled along with stress or anxiety. These triggers mirrored those described by food insecure (FIS) participants Financial resources may be available, but physical access is challenged e.g., moving to an area with limited public transport infrastructure/no car	Triggers/trigger risks are constantly in the background.
			Budget/financial/income triggers: shocks
			Cost of Living expenses and bill shocks: utilities and seasonal fluctuations. E.g., an increase in child care fees and unresponsive government support
			Changes to household composition: these may be short or long-term but consequential impacts are felt. E.g., addition of a child or family member (adult child/sibling)
			Change in relationship status: divorce
			Budget stress of trying to shop in bulk or shop for specials: trying to plan ahead.
**External triggers**	Perceived fluctuations in cost of food	*	Households may not have the financial resources to weather food cost changes, especially when this is added to other internal triggers.
	Physical access to food shops, availability beyond the Coles/Woolworths-type supermarkets, the preference for local shopping		
**Theme 4: Assets amplified: juggling and applying management strategies as required**			
**Households transform**	Assets are enacted in both households but at different levels (amplification effect)	Budget/shopping management assets are present, but are not or are rarely amplified to the extent of food insecure households.	Asset pooling and juggling across the households. Often, it is just the assets from the household gatekeeper wearing the stress and strain. Amplification of transformation of assets
**assets into action**			Assets used in all situations at home: day-to-day, entertaining at home, and eating out/purchase of takeaway food
**Transform and adapt assets with external support**	Both may receive financial support from Government benefits: Family Tax Benefit, Child Care Rebate, study assistance. Households attend community-based activities: gardens, farmers markets, or similar (food source, social) but often for a different purpose.	May have the social support assets but serve a different purpose than in FIS households. Not used as a food access means.	Households may require the assets that are transformed through social/financial support: community, family, or friends, and not through welfare/food relief agencies. Issues of inability to access, and pride; there are those who are in greater need. Households rely on grandparents to pay for activities, bring food, or ‘shout’ lunch in food court
**Theme 5: The consequences and emotional rollercoaster of food access and provision**			
**Stress and strain matched with give and take**	Attempts to protect children if food is scarce Frustrations in both households: cost of food, availability of food, marketing of food	Some food-secure households that have experienced food insecurity or have been at risk of food security in their lifetime reflected on the level of impact of the experience and the strain, and how this has shaped their desire to not experience this again: stress, embarrassment.	Often significant compromise on food quality, quantity, and nutrition: these are constantly amplified across households compared to food secure (FS) households. Compromises may be limited to one person in the household: the food gatekeeper.
			Guilt associated with compromises, especially if other household members (children) are affected.
			The relentless, constant stresses of making ends meet: the load of this, the potential for allostatic load, and impacts on physical, social, and emotional wellbeing. This is amplified in these households.
			Social consequences: the compromise that is made to these opportunities and potential repercussions to self and household budgets.
**4R’s: Resilience, Respect, Resourceful, and Responsible**	Pride/respect in strategies and skills that a household may possess, especially relating to food procurement, cooking, and sharing. Resilience/Respect/Resourcefulness Responsibility present in all households, but greater in FIS households	Present and in action, but the intensity may vary across and within households	Present in FIS households, but is greatest for the food/household gatekeeper: amplification effect

* No additional difference noted.

## Supplementary Materials

The following are available online at http://www.mdpi.com/1660-4601/15/10/2206/s1, Table S1: Quantitative United States Department of Agriculture Food Security Survey Module (18 items) and Qualitative Question Logic.
