# Supplementary material for: Walking the Food Security Tightrope—Exploring the Experiences of Low-to-Middle Income Melbourne Households
Source: Int J Environ Res Public Health. 2018 Oct 10;15(10):2206. doi: 10.3390/ijerph15102206 (PMC6210237; doi:10.3390/ijerph15102206)
Supplement: Supplementary file 1 [file ijerph-15-02206-s001.pdf]

Supplementary table S1 for manuscript titled: Walking the food security tightrope – exploring the experiences of low-to-middle income Melbourne households

Table S1: Quantitative United States Department of Agriculture Food Security Survey Module (18item) and Qualitative Question Logic

USDA FSSM Question Item	Qualitative Question Logic	Question Areas*
**Adult specific items**		
We worried whether our food would run out before we got money to buy more.	Worry, stress – what was the level of concern? Consequences?	Areas 2, 3
The food that we bought just didn't last and we didn't have money to get more.	Consequences? Reason for inability to access food? How did households attempt to make food last? Skills and knowledge-assets?	Areas 2,3,4
We couldn't afford to eat balanced meals.	Consequence? Compromise of nutrition? Were there specific foods? Food knowledge?	Areas 1,2,3
In the last 12 months, did you or other adults in the household ever cut the size of your meals or skip meals because there wasn't enough money for food?<br><br>Frequency response: In the last 12 months, did you or other adults in the household ever cut the size of your meals or skip meals because there wasn't enough money for food?<br><br>Almost every month/some months/not every month	Consequences? To what degree did these happen? Impact for the household? Are there protective and coping strategies before it reaches this point?	Areas 1,2,3,4
In the last 12 months, did you ever eat less than you felt you should because there wasn't enough money for food?	Consequences? Specific foods? Frequency? Did this affect other members of the household? How are food decisions made?	Areas 1,2,3,4
In the last 12 months, were you ever hungry, but didn't eat, because there wasn't enough money for food?	Consequences? Frequency? What was the extent of financial triggers? What were the decisions at this point?	Areas 1,2,3,4

USDA FSSM Question Item	Qualitative Question Logic	Question Areas*
In the last 12 months, did you lose weight because there wasn't enough money for food?<br><br>In the last 12 months did you or other adults in your household ever not eat for a whole day because there wasn't enough money for food?<br><br>Frequency response: In the last 12 months did you or other adults in your household ever not eat for a whole day because there wasn't enough money for food?<br><br>Almost every month/some months not every month	Are there protective and coping strategies before it reaches this point?	
**Children specific items**		
We relied on only a few kinds of low-cost food to feed our children because we were running out of money to buy food.<br><br>'We couldn't feed our children a balanced meal, because we couldn't afford that<br><br>'The children were not eating enough because we just couldn't afford enough food.'<br><br>In the last 12 months, did you ever cut the size of any of the children's meals because there wasn't enough money for food?<br><br>In the last 12 months, did you ever cut the size of any of the children's meals because there wasn't enough money for food?<br><br>In the last 12 months, were the children ever hungry but you just couldn't afford more food?	Consequences? Decisions? Factors impacting on food protective and coping strategies?	Areas 1,2,3,4

USDA FSSM Question Item	Qualitative Question Logic	Question Areas*
In the last 12 months, did any of the children ever skip a meal because there wasn't enough money for food?		
Frequency response		
In the last 12 months, did any of the children ever skip a meal because there wasn't enough money for food?		
In the last 12 months did any of the children ever not eat for a whole day because there wasn't enough money for food?		

*Question Areas: 1. accessing food and food choices for the household, 2. factors impacting on food for the household, 3. consequences when sufficient food quantity and preferred foods cannot be accessed, 4. coping and protective strategies - asset exploration
